# Use of Mental Health Services for Patients Diagnosed with Major Depressive Disorders in Primary Care

**DOI:** 10.3390/ijerph18030885

**Published:** 2021-01-20

**Authors:** Laura González-Suñer, Cristina Carbonell-Duacastella, Ignacio Aznar-Lou, Maria Rubio-Valera, Maria Iglesias-González, Maria Teresa Peñarrubia-María, Montserrat Gil-Girbau, Antoni Serrano-Blanco

**Affiliations:** 1 Parc Sanitari Sant Joan de Déu, Sant Boi de Llobregat, 08830 Barcelona, Spain; l.gonzalez@pssjd.org (L.G.-S.); aserrano@pssjd.org (A.S.-B.); 2 Teaching, Research & Innovation Unit, Institut de Recerca Sant Joan de Déu, Sant Boi de Llobregat, 08830 Barcelona, Spain; c.carbonell@pssjd.org (C.C.-D.); mrubio@pssjd.org (M.R.-V.); m.gil@pssjd.org (M.G.-G.); 3 Instituto de Salud Carlos III, Centro de Investigación Biomédica en Red de Epidemiología y Salud Pública (CIBERESP), 28029 Madrid, Spain; maitepenarrubia@gmail.com; 4 Hospital Universitari Germans Trias i Pujol, 08916 Badalona, Spain; m.iglesias.gonzalez@gmail.com; 5 Institut Català de la Salut i Institut d’Investigació en Atenció Primària Jordi Gol (IDIAP Jordi Gol), 08006 Barcelona, Spain

**Keywords:** major depressive disorder, mental health services, family physician, primary care

## Abstract

Major depressive disorder (MDD) is one of the most disabling diseases worldwide, generating high use of health services. Previous studies have shown that Mental Health Services (MHS) use is associated with patient and Family Physician (FP) factors. The aim of this study was to investigate MHS use in a naturalistic sample of MDD outpatients and the factors influencing use of services in specialized psychiatric care, to know the natural mental healthcare pathway. Non-randomized clinical trial including newly depressed Primary Care (PC) patients (*n* = 263) with a 12-month follow-up (from 2013 to 2015). Patient sociodemographic variables were assessed along with clinical variables (mental disorder diagnosis, severity of depression or anxiety, quality of life, disability, beliefs about illness and medication). FP (*n* = 53) variables were also evaluated. A multilevel logistic regression analysis was performed to assess factors associated with public or private MHS use. Subjects were clustered by FP. Having previously used MHS was associated with the use of MHS. The use of public MHS was associated with worse perception of quality of life. No other sociodemographic, clinical, nor FP variables were associated with the use of MHS. Patient self-perception is a factor that influences the use of services, in addition to having used them before. This is in line with Value-Based Healthcare, which propose to put the focus on the patient, who is the one who must define which health outcomes are relevant to him.

## 1. Background

Major depressive disorder (MDD) is a highly prevalent mental disorder [1], associated with lower quality of life and social functioning, higher mortality, and greater burden due to high prevalence, relapse rates, and a tendency towards chronification [2,3]. Taken together, these factors lead to greater use of health services, reduced productivity, and more days of sick leave. Despite this, rates of recognition and adequate treatment are very low [4,5]. Adequate recognition of patients with depression by the Family Physician (FP) may be as low as 22%, and, of those diagnosed, three in every four were “false positives”, with rates of treatment adequacy between 39.35% and 54.91% [4,5,6]. This results in worse short-term outcomes and represents a major public health concern [7,8].

Despite the fact that a high percentage of cases with first episodes of depression in the general population recover on their own [9], findings show that those who have not recovered in the first months, have high risks of adverse outcomes [10], so it is important to examine which factors are related to the use or not of mental health services, where adequacy of diagnosis and effectiveness of treatment is higher, and the proportion of patients receiving minimally adequate care is superior (38.6% compared with 12.8%) to that in Primary Care (PC) [5,11].

There are several studies that have attempted to determine which features distinguish those consulting Mental Health Services (MHS) from those who do not. The results are inconsistent and often contradictory. Some found no differences in sociodemographic variables [12]. Other studies found that being female, not being married, having a higher level of education and being younger than 65 years of age are factors that increase the likelihood of consultation [11,13,14,15,16,17,18,19,20,21]. Regarding clinical variables, some studies have related using MHS and having more severe, recurrent, or chronic depression, poor perceived physical health, and more than one mental disorder, especially anxiety disorders [5,11,13,14,16,18,20]. These contradictory and inconsistent results could be telling us about the lack of clear criteria for MHS access, and the absence of criteria that guarantee the right to be treated in those cases that need it most (due to severity, comorbidity, suicidal thoughts). 

It is important to determine not only the factors related to the patient, but also those related to the FP, since in the Spanish PC public system the FP acts as the main gatekeeper to the health system and decides which patients are referred to Mental Health Services (MHS) according to their clinical criteria. Each individual has an assigned FP, so we can assume that the characteristics of the FP (their knowledge of mental health, their beliefs about depression, their training, etc.) may be factors influencing the approach to the case, and therefore in its referral or not to MHS. However, to our knowledge, the factors associated with FP have scarcely been studied, and the results are contradictory. A study carried out in Hong Kong points to possible influences of FP characteristics on prognosis (age, public sector, and training in Family Medicine), but without finding statistically significant associations [22]. Another study published in 2018 also points to the influence of some FP characteristics [23], but not other studies [13].

However, we also know that resources in Spanish MHS are scarce or less available than in other European countries [24], and the access to these services is not only influenced by sociodemographic or clinical factors, but also by cultural factors, such as stigma or mental health literacy, as well as organizational factors and national mental health policies, such as the FP-population ratio, effective availability of mental health specialists, existence or absence of a gatekeeping system, how mental health costs are covered, or the relationship between PC and MHS [25,26]. In Catalonia, the north eastern Spanish autonomous community, a widespread private health system exists with a long tradition of co-existence with the public sector. The use of public or private MHS could be socioeconomic and contextual factors. 

The aim of this study was to investigate MHS use in a naturalistic sample of MDD outpatients and the factors influencing use of services in specialized psychiatric care, to know the natural mental healthcare pathway.

## 2. Methods

### 2.1. Study Design and Participants

This study was conducted as part of a larger naturalistic prospective controlled trial (INFAP study [27]). The aim of this study was to compare the cost-effectiveness of antidepressants versus active monitoring in patients with a new Major Depressive Episode (MDE) in the PC setting. 

The study was conducted from 2013 to 2015 in 12 health care centers in the metropolitan area of Barcelona (Catalonia, Spain). In Spain, the public health system is taxpayer-funded, universal for residents and foreign nationals and free at the point of use (with some exceptions, such as medication). There are over 300 PC centers located throughout the region, and access to specialized services requires a referral by the FP. These services are compatible with the use of private health services, so we distinguished between overall use of MHS (public and private) and public MHS, specifically.

Patients were included in the study by the FP if they were ≥18 years old; with a diagnosis of a new mild-moderate MDE (in contrast to chronic conditions, such as dysthymia) according to the FP’s clinical judgment; had not taken antidepressant medication in the previous 60 days or antipsychotics, lithium, or antiepileptics in the previous six months; and without psychotic or bipolar disorder, history of drug abuse, or dependency or cognitive impairment. Patients with a new severe MDE were excluded since, following guideline recommendations, the main focus of the INFAP study was comparison of the effectiveness of Active Monitoring (AM) and Antidepressants in treating mild-moderate MDE in PC.

Once a patient was newly diagnosed with a MDE, FPs offered patients to participate into the study, informed them of the study aims and obtained signed consent. Subsequently, FPs referred patients to an independent, trained psychologist for the first assessment. Patients were assigned to AM or antidepressant according to FPs’ clinical judgment. Patients decision on participating in the study did not influence the group assignment.

Previous experiences in similar settings and population suggested to include 150 patients per arm [27].

The study was approved by the Clinical Research Ethics Committee at Sant Joan de Déu Foundation (CEIC Fundació SJD; Reference Number: EPA-24-12) and the Clinical Research Ethics Committee at the Jordi Gol Research Institute (CEIC IDIAP; Reference Number: 5013–002). This article arises from the secondary data analysis of the INFAP study, which may affect methodological aspects.

### 2.2. Outcomes

#### 2.2.1. Patient Variables

The sociodemographic questionnaire requested information on gender, age, civil status, cohabitation, education, and working status.

The research version of the Structured Clinical Interview for Diagnostic and Statistical Manual of Mental Disorder (DSM) IV Axis I Disorders (SCID-I) was used to assess clinical diagnosis of mood and anxiety disorders, according to DSM-IV diagnostic criteria [28]. Any patient with a diagnosis of MDD according to the SCID-I, in addition to another anxiety or adjustment disorder according to the same scale, was considered as having comorbidity with anxiety disorders.

The Patient Health Questionnaire 9-item depression module (PHQ-9) was used to evaluate the severity of depressive disorder [29,30,31]. It is a scale with items scored from 0 (never) to 3 (nearly every day) on nine symptoms of depression, with a total score ranging from 0 (no depressive symptoms) to 27 (all symptoms occurring daily). Scores from 0 to 4 correspond to minimal symptoms; 5 to 9: mild; 10 to 14: moderate; 15 to 19: moderately severe; and 20 to 27: severe. This scale showed a Cronbach alfa of 0.86. Given the importance of autolytic ideation (suicidal thoughts) as an element of severity influencing use of health services, which has been associated with MHS in previous studies [13], we decided to evaluate it through one of the items on this scale.

The Beck Anxiety Inventory (BAI) was used to assess the severity of anxiety. It is composed of twenty-one self-reported items scored from 0 (not at all) to 3 (severely). Summed scores range from 0 to 63 [32]. Higher scores mean more severe anxiety symptomatology. The score can be categorized: from 0 to 9: normal anxiety; 10 to 18: mild; 19 to 29: moderate; and 30 to 63: severe. This scale showed a Cronbach alfa of 0.91.

The Spanish version of the EuroQol-5D (EQ-5D) was used to evaluate health-care related quality of life [33,34,35]. The EQ-5D evaluates five health domains: mobility, self-care, usual activities, pain/discomfort, and anxiety/depression, on three levels of severity (no problems, some problems, extreme problems). It generates 245 possible health states which correspond to a tariff [36]. Value equal to 1 represents the best health state, and value equal to 0 represents being dead; however, there may be also negative values that correspond to health states perceived as worse than death. These health states have utility scores assigned by using the readily available Spanish population tariffs. The EQ-5D also includes a visual analogue scale (VAS), with a score between 0 (the worst imaginable health state) and 100 (the best imaginable health state).

The interviewer-administered 12-item version of the World Health Organization Disability Assessment Schedule (WHO-DAS 2.0) [37,38,39] was used to assess disability. The WHO-DAS 2.0 assesses the level of difficulty experienced in the previous month by the respondents. Items are scored using a 5-point scale (none = 1, mild = 2, moderate = 3, severe = 4, extreme/cannot do = 5). The total score ranges, based on WHO instructions, are from 0 to 100, with higher scores reflecting greater disability. This scale showed a Cronbach alfa of 0.83.

The Brief Illness Perception Questionnaire (BIPQ) was used to evaluate perception of illness [40,41], with 9 items evaluating the following dimensions: identity, consequences, timeline, personal control, treatment control, concern, understanding, and emotional representations. Total score ranges from 0 to 90. Higher scores mean worse beliefs about depression. This scale showed a Cronbach alfa of 0.80. 

Patients were included in active monitoring or antidepressant groups depending on the clinical criteria of their FP. Following the Catalan Clinical Practice Guideline, active monitoring consisted of a first follow-up visit within the next 15 days, as well as consideration of a specific brief psychosocial treatment of between 6 and 8 sessions. In case of no improvement, the FP can initiate antidepressants. 

Detailed information on instrument validation for the Spanish population may be found in the study protocol [27].

#### 2.2.2. Use of MHS

Client Service Receipt Inventory (CSRI) was used to assess the use of MHS resources in the 12 months prior to and following the diagnosis [42], specifically items related to the use of public and private psychiatric and psychological services. Any patient who had used any of the above resources, at least once, was considered as mental health care service user. A similar approach was followed for public users.

#### 2.2.3. FP Variables

FP variables were evaluated through a paper-and-pencil self-reported questionnaire. Sociodemographic questionnaire information included: gender, age, resident’s tutor, specialist in family medicine, average time they used per consultation, time working in the current position, mental health interest, communication with mental health team, support from mental health team, comfort with the use of antidepressants, and attitudes towards depression assessed with the Depression Attitudes Questionnaire (DAQ) adapted into Spanish [43]. The DAQ comprises 20 items scored from 0 (completely disagree) to 4 (completely agree), with a total score ranging between 0 and 80; higher scores mean better attitudes toward Major Depression. This scale showed a Cronbach alfa of 0.81.

### 2.3. Statistical Analysis

The dependent variable was being an MHS user. A multilevel logistic regression analysis was performed to determine which factors were associated with the use of MHS. Subjects were clustered by FP. Intraclass correlation coefficient were <0.1. Since previous use of MHS was expected to strongly influence future use, we decided to include this variable in the basic model. In order to select other relevant variables, we first conducted logistic regression models where the dependent variable was the use of MHS in the 12 months following the diagnosis and the independent variable was each of the factors that had the potential to predict use of MHS adjusted by use of MHS in the previous 12 months (three-variable model). Those factors with a p-value lower than 0.2 were included in the multilevel multivariate logistic regression model [44]. We calculated Odds Ratio (OR) and 95% confidence interval.

The same approach was followed to evaluate factors associated with the use of public MHS. 

The proportion of missing data in all values was lower than 26%. Variables with missing data were imputed using multiple imputations by chained equation. All variables that were associated with the probability of missingness, and those that were used in the posterior analyses were included in the imputation model. Number of imputations was calculated using a rule of thumb with respect to the fraction of missing information [45]. We imputed 20 datasets.

Stata MP 13 (College Station, TX, USA) for Windows was used to perform all the analyses.

## 3. Results

### 3.1. Sociodemographic and Clinical Characteristics of the Sample

Patient and FP sample characteristics are presented in Table 1. Of 263 patients, 81% were women with a mean age of 49; 31% met criteria for MDE according to the SCID-I, and this percentage increased to 43% by including patients with comorbid anxiety disorder. The average severity of depression symptomatology score corresponded to moderately severe depression and the severity of anxiety score between mild and moderate. 

Of the sample of 53 FPs, most were specialists in general medicine (83%), and they had a mental health interest score of 8 out of 10.

### 3.2. Factors Associated with Use of Public and/or Private MHS

Of 263 patients, 27% had consulted MHS in the 12 months following the diagnosis: 19% used public MHS, 15% used private MHS, and 27% used any MHS. In the previous 12 months, 3% had used public MHS, 6% private MHS, and 8% any MHS. 

Factors associated with the use of MHS are shown in Table 2. The reference population was all those patients who did not use MHS. In the three-variable analysis, patient variables associated with a lower probability of using MHS were being treated with Active Monitoring (OR = 0.49), being older (OR = 0.97) and having poorer quality of life according to the VAS (OR = 0.97). Patient variables related to using MHS in the 12 months after the diagnosis were having used MHS in the previous year (OR = 6.20), higher severity of anxiety (OR = 1.04) and worse beliefs about the illness (OR = 1.04). Regarding FP variables, being treated by a resident’s tutor FP decreased the probability of using MHS (OR = 0.39), while those patients whose FP had bad communication with the mental health team had a higher probability of using MHS (regular communication OR = 0.14; good communication OR = 0.10; very good communication OR = 0.04). 

In the multivariate analysis, only having used MHS in the previous year was associated with the use of MHS in the following 12 months (OR = 5.02). No FP factor maintained the association. 

### 3.3. Factors Associated with Use of Public MHS

Table 3 shows factors associated with the use of Public MHS. The reference population was all those patients who did not use Public MHS. In the three variable analysis, a level of primary education, lower quality of life scored by VAS, worse beliefs about illness, and greater severity of anxiety were associated with the use of public MHS (OR = 0.09, OR = 0.97, OR = 1.06, and OR = 1.04, respectively). Regarding FP characteristics, bad communication with mental health team increased the odds of using public MHS (regular communication OR = 0.06; good communication OR = 0.07; very good communication OR = 0.01). 

In the multivariate model, only quality of life scored through the VAS maintained the association (OR = 0.97). No FP factor maintained the association. 

## 4. Discussion

We found that only 27% of individuals diagnosed with a new MDE by their FP used MHS within 12 months of study commencement. As shown in previous studies, only having consulted MHS in the previous year increased the odds of consulting specialized services after the diagnosis of new MDE [46]. This probably occurs because the patient asks for the referral or the FP could raise this option earlier if the patient had attended MHS previously. 

No associations were found between sociodemographic variables and MHS use, in contrast with other studies, which found influence of gender, age, or educational level in the decision to consult MHS [11,13,14,15,16,17,18,19,20,21]. As shown by Boerema et al. in 2017 [47], this fact may be a positive result, since receiving help should not be based on factors, such as age, gender, or educational level, and would be consistent with the universality that the national health system in Spain defends. 

Although it is true that different sociodemographic variables are also associated with specific and different needs (women vs. men; young vs. older patients; higher or lower educational level, etc.), not finding differences in our sample could also be a symptom of our incapacity of reaching target groups, and it is showing us possible "gaps" in care, and the importance of developing specific programs adapted to the needs of each specific group (facilitate knowledge of existing resources, the access to them, and adapt treatment strategies).

Regarding clinical variables, no associations were found either, in contrast with previous studies which found influence of severity of depression or the presence of autolytic ideation, as well as comorbidity with anxiety disorders [5,11,13,14,16,18,20]. This is due to INFAP study recruitment criteria. The main aim of the INFAP study was to determine the cost-effectiveness of pharmacological versus non-pharmacological interventions used to treat those having a new episode of mild-moderate MDE and being treated in PC. National and international clinical guidelines [48,49,50] suggest treating these patients with non-pharmacological interventions and, additionally, there is a health policy that encourages FP to treat those patients in PC in Catalonia. Thus, compared with previous papers, less severe MDE patients were included in the INFAP study, and no clinical variables influenced the FP to refer the patient to MHS. 

Worse health related quality of life measured through the VAS was found among those patients attending public MHS compared with those not attending public MHS. Data showed that the EQ-5D, based on specific constructs, was not associated with the use of MHS, but the VAS, which is based on overall self-perception of quality of life, was. Therefore, self-perception of patients’ quality of life was more influential than the actual severity of symptoms. This coincides with the results obtained in a previous study [23]. According to Andersen, how people view their own general health, how they experience symptoms, and whether they believe their problems are of sufficient magnitude to seek professional help should be considered [51]. Previous studies already indicated perceived need as a key variable in better understanding of the use of services and therapeutic compliance [13,23]. As such, we believe it is important to take this subjectivity into account.

No other variables, sociodemographic, clinical, nor related to FP, were predictive of consulting MHS. The fact that FP’s variables were not associated with the use of MHS could be due to the small size of the sample (only 53 FPS) and be biased by the FPs selection, which was voluntary and, therefore, based on their interest in depression and mental health, in general. However, it can also be considered something positive, if we think that the referral or not to MHS is based exclusively on patients and their perceived needs, and not so much on FP’s variables (which could contribute to unequal treatment between patients depending on which FP is assigned to each one). Despite this, our data does not allow us to draw conclusions in this regard, and a broader and more in-depth study is necessary.

There are other variables, such as cultural factors, stigma, mental health knowledge, and organizational and national mental health policies [19,25,26], which can influence and promote these inconsistent and contradictory results in different countries and differing health systems, in which resources and requirements to access health services vary greatly and can significantly affect the results. Specifically, in Spain, there is a more positive attitude towards seeking mental health care, greater confidence in the treatments provided by mental health professionals [52], and stigma may be less prevalent than in other European countries [53,54], which could lead to more widely extended help-seeking behavior. However, Spain is also a country in which public psychiatrists, psychologists, and MHS in general are less available than in many other countries with the same developmental level, according to the WHO Mental Health Atlas, 2017 [24]. This could have influenced the results and could leave target patients unattended, along with increasing the use of private MHS or medical insurance in those patients with greater purchasing power, etc. More research is needed to determine the influence of all these variables and how they can affect the results. 

For future studies, it would be important to collect a larger sample, including those patients with more severe depression, in order to see its influence on the results. It would also be important to analyze what blocks target patients’ access to MHS (stigma, difficulty of access, available resources, difficulty of referral from the PC, etc.) and to improve the management of less serious cases in PC by FPs to facilitate and increase the adequacy of referrals.

All this may imply changes in FP training and more effective development and dissemination of clinical guidelines, as well as taking into account organizational variables and national mental health policies, such as the need to improve communication and collaboration between PC and MHS, and augment resources and financial coverage for MHS.

Given the multitude of variables that can influence help-seeking behaviors and access to health resources (sociodemographic, clinical, cultural, political, etc.), it is important to expand this study in the future, taking into account these variables, as well as overcoming the limitations of our study, such as the exclusion of those cases with severe MDE.

### Strengths and Weaknesses

As strengths, the naturalistic context of the study allows us to view the health care pathways of patients within different care services (general and specialized) without influencing them. Additionally, the inclusion of FP variables, as potential predictors of care received by patients, highlights the need for further studies in this regard.

In interpreting these results, a number of weaknesses should be considered. The most important has to do with the fact that this analysis emerges as a secondary analysis of another main study (INFAP study), having excluded severe cases of MDE, and including a sample of patients with a new mild-moderate MDE, which is not representative of all depressed patients receiving care PC and could limit our conclusions to all depressed patients in PC. To this is added the fact that data collection ended 5 years ago, which may also mean that some of the results cannot be extrapolated to the current reality, although we consider that the social and psychopathological conditions of the population have not been modified a lot in recent years, with regard to the approach to patients with MDE in PC.

In addition, the small sample size, along with the FP selection, i.e., including FPs that decided to participate in the study on a voluntary basis, which may be indicative of a greater interest in mental health area but not necessarily representative of average FP behavior, could be considered as limitations. 

## 5. Conclusions

Referring to MHS (public or not) depends not so much on the severity of symptomatology, but on the patient’s self-perception, highlighting the importance of the patient’s previous experience of distress. It also depends on whether the patient has previously consulted MHS. No other variable appeared to influence the use of MHS. 

It seems that, currently, in Spain, subjective criteria prevail in the decision algorithm to refer a patient with MDE to MHS over objective criteria, such as sociodemographic or clinical aspects. This allows us to see the importance of attending to the patient’s subjectivity and can be in line with Value-Based Healthcare, which propose to change the conceptual framework and put the focus on the health result and not so much on the process. It would be to go from attending to "what is done", to attending to "what is obtained", putting the focus on the patient, who is the one who must define which health outcomes are relevant to him [55]. In this sense, previous studies talk about the importance of patients’ perceived helpfulness of depression treatment and how this can significantly influence the use of MHS [56,57].

## Figures and Tables

**Table 1:** Characteristics of patients and Family Phyisicans at baseline.

Patient Characteristics (*n* = 263)	N	%
*Sociodemographic characteristics*		
Female	213	81.0
Age (mean ± SD)	48.93 ± 15.4	
Marital status Single Married/In couple Separated/Divorced Widow(er) Cohabitation (living with someone vs. alone)	41 160 45 17 224	15.6 60.8 17.1 6.5 85.2
Education No formal education Primary Completed primary education Secondary University Not known/Not answered	19 45 71 91 36 1	7.2 17.1 27.0 34.6 13.7 0.4
Working status Active worker Not paid worker (including homemaker, student) Worker on medical leave Pensioner unemployed Non-pensioner unemployed Retired	76 32 49 35 41 30	28.9 12.2 18.6 13.3 15.6 11.4
*Clinical state*		
Clinical diagnosis according to SCID * Major depression according to DSM-IV criteria alone Major depression + anxiety disorder according to DSM-IV criteria Previous Major depression according to DSM-IV criteria	81 33 41	30.8 12.6 15.6
Severity of depression symptomatology (PHQ-9); scale:0–27 (mean ± SD)	16.2 ± 5.4	
Suicidal ideation (item from PHQ-9) * Not at all Several days More than half the days Nearly every day	154 67 17 23	59.3 25.5 6.5 8.7
Severity of anxiety (BAI); scale:0–63 (mean ± SD)	18.2 ± 10.8	
EuroQoL-5D; scale:-0.0757–1.0000 (mean ± SD)	0.59 ± 0.2	
EuroQoL-5D-VAS; scale:0–100 (mean ± SD)	44.9 ± 20.1	
Disability (WHO-DAS 2.0); scale:0–100 (mean ± SD)	37.3 ± 18.4	
Beliefs about illness (BIPQ); scale:0–90 (mean ± SD)	47.3 ± 9.5	
FP characteristics (*n* = 53)	**N**	**%**
Female	45	84.9
Age (mean ± SD)	44.3 ± 6.7	
Resident’s tutor	22	41.5
Specialist in family medicine	44	83.0
Mental Health interest; scale:0–10 (mean ± SD)	7.9 ± 1.6	
Communication with mental health team (categorized) Very bad Bad Regular Good Very Good	2 14 30 7	No sample 3.8 26.4 56.6 13.2
Comfort with use of antidepressants (categorized) Very uncomfortable Uncomfortable Neither comfortable nor uncomfortable Comfortable Very comfortable	9 37 7	No sample No sample 17.0 69.8 13.2
Attitudes towards depression (mean ± SD)	42.3 ± 5.5	

* In this scale, the total sample was 261, since there were two missing at baseline.

**Table 2:** Factors associated with use of public and/or private Mental Health Services (MHS) based on three variables and multivariate imputed multilevel logistic regression models *.

	Three Variable Analyses		Multivariate Analyses	
Patient Characteristics (*n* = 263)	OR	95% CI	OR	95% CI
Treatment without antidepressants (active monitoring)	**0.49**	**0.25–0.98**	0.54	0.23–1.26
Female	0.66	0.30–1.44	1.03	0.38–2.83
Age	**0.97**	**0.95–1.00**	0.98	0.94–1.01
Use of MHS prior to study commencement	**6.20**	**2.12–18.17**	**5.02**	**1.34–18.84**
Education No formal education Primary Completed primary education Secondary University	Ref. 0.26 0.45 1.06 1.35	- 0.05–1.26 0.13–1.61 0.32–3.51 0.37–4.98	Ref. 0.43 0.44 0.75 1.03	- 0.07–2.56 0.07–2.74 0.13–4.46 0.15–7.12
Working status Active worker Not paid worker (including homemaker, student) Worker on medical leave Pensioner unemployed Non-Pensioner unemployed Retired	Ref. 0.60 1.57 0.68 0.70 0.33	- 0.19–1.91 0.66–3.73 0.21–2.24 0.24–2.08 0.08–1.30	Ref. 0.79 1.29 0.64 0.41 1.20	- 0.19–3.18 0.45–3.65 0.17–2.40 0.10–1.72 0.17–8.34
Clinical state **				
Severity of depression symptomatology (PHQ-9)	1.06	0.99–1.14	1.01	0.91–1.12
Severity of anxiety (BAI)	**1.04**	**1.01–1.07**	1.01	0.97–1.05
EuroQoL-5D-VAS	**0.97**	**0.96–0.99**	0.98	0.96–1.00
Disability (WHO-DAS 2.0)	1.02	1.00–1.04	1.00	0.98–1.03
Beliefs about illness (BIPQ)	**1.04**	**1.00–1.09**	1.02	0.96–1.07
**FP characteristics**	**OR**	**95% IC**	**OR**	**95% IC**
Female	2.05	0.63–6.68	1.66	0.36–7.58
Specialist in family medicine	0.47	0.19–1.05	0.82	0.30–2.26
Resident’s tutor	**0.39**	**0.20–0.76**	0.55	0.22–1.34
Communication with mental health team (categorized)				
Bad	Ref.	-	Ref.	-
Regular	0.14	0.01–1.59	0.17	0.01–2.66
Good	**0.10**	**0.01–1.00**	0.13	0.01–1.80
Very good	**0.04**	**0.00–0.52**	0.05	0.00–1.05
Comfort with use of antidepressants (categorized) Neither comfortable nor uncomfortable Comfortable Very comfortable	Ref. 0.88 0.39	- 0.37–2.07 0.10–1.44	Ref. 1.17 0.91	- 0.38–3.66 0.17–4.77
Attitudes towards depression	1.04	0.98–1.10	1.06	0.97–1.16

Statistically significant association (95%) is highlighted in bold; * The reference population was all those patients who did not use any MHS; ** Only variables with a *p*-value <0.2 were included in the multilevel multivariate regression model.

**Table 3:** Factors associated with use public Mental Health Services (MHS) based on three variable and multivariate imputed multilevel logistic regression models *.

	Three Variable Analyses		Multivariate Analyses	
Patient Characteristics (*n* = 263)	OR	95% CI	OR	95% CI
Group (Active Monitoring vs. Antidepressants)	0.54	0.24–1.19	0.78	0.30–2.07
Age	0.98	0.96–1.01	1.00	0.96–1.04
Use of Public MHS prior to study commencement	5.47	0.91–33.01	5.75	0.66–50.01
Education No formal education Primary Completed primary education Secondary University	Ref. **0.09** 0.25 0.78 0.66	- **0.01–0.83** 0.06–1.06 0.22–2.72 0.15–2.90	Ref. 0.14 0.27 0.69 0.91	- 0.01–1.75 0.04–1.76 0.11–4.19 0.13–6.23
Clinical state**				
Severity of depression symptomatology (PHQ-9)	1.07	0.99–1.16	0.98	0.86–1.12
Severity of anxiety (BAI)	**1.04**	**1.00–1.07**	1.00	0.96–1.05
EuroQoL-5D-VAS	**0.97**	**0.94–0.99**	**0.97**	**0.94–1.00**
Disability (WHO-DAS 2.0)	1.02	1.00–1.04	1.00	0.97–1.03
Beliefs about illness (BIPQ)	**1.06**	**1.01–1.11**	1.05	0.99–1.12
**FP characteristics**	**OR**	**95% IC**	**OR**	**95% IC**
Resident’s tutor	0.51	0.22–1.18	0.63	0.22–1.81
Communication with mental health team (categorized)				
Bad	Ref.	-	Ref.	-
Regular	**0.06**	**0.00–0.71**	0.06	0.00–1.32
Good	**0.07**	**0.01–0.80**	0.09	0.00–1.65
Very good	**0.01**	**0.00–0.30**	0.00	0.00–0.00
Attitudes towards depression	1.06	0.99–1.13	1.08	0.99–1.18

Statistically significant association (95%) is highlighted in bold; * The reference population was all those patients who did not use Public MHS. ** Only variables with a *p*-value < 0.2 were included in the multilevel multivariate regression model.

## Data Availability

Please refer to suggested Data Availability Statements in section “MDPI Research Data Policies” at https://www.mdpi.com/ethics.
